# Calciphylaxis in the Setting of Acute Kidney Injury

**DOI:** 10.7759/cureus.82018

**Published:** 2025-04-10

**Authors:** Priyank Desai, Claudville Derby, Martine A Cioffi, Hanady Zainah, Mark Majed Samarneh, Maria Laureana Santos-Zabala

**Affiliations:** 1 Internal Medicine, St. John's Riverside Hospital, Yonkers, USA; 2 Nephrology, St. John's Riverside Hospital, Yonkers, USA; 3 Pathology, St. John's Riverside Hospital, Yonkers, USA

**Keywords:** arterial calcification, calciphylaxis, end-stage renal disease (esrd), sodium thiosulfate, warfarin

## Abstract

Calciphylaxis, medically referred to as calcemic uremic arteriolopathy, stands as a rare and life-threatening dermatological condition that frequently emerges as a complication of chronic kidney disease (CKD), primarily affecting patients with end-stage renal disease (ESRD), who are typically undergoing dialysis. This condition is characterized by the development of cutaneous ischemic infarcts, originating from blood vessel obstructions within the subcutaneous fat and dermis. It is imperative to recognize that calciphylaxis is associated with an alarmingly high mortality rate, with more than 50% of afflicted individuals succumbing within a year, often due to complications like sepsis. Intriguingly, there have been relatively few documented instances of calcemic uremic arteriolopathy occurring in non-ESRD patients.

In this paper, we undertake an extensive examination of calciphylaxis, delving into its pathophysiology, diverse clinical presentation, and the array of treatment modalities available. To illustrate these facets, we present a case report featuring a man afflicted with calciphylaxis in the setting of acute kidney injury (AKI), thus providing valuable insights into the atypical condition of this already rare disease. Given the scarcity of literature pertaining to calciphylaxis in patients with AKI, this case study aims to contribute valuable insight into potential factors underlying calcemic uremic arteriolopathy.

## Introduction

Calciphylaxis, a condition known for its occlusive nature affecting cutaneous blood vessels, is characterized by the blockage of these vessels and the subsequent development of ischemic injuries. This occlusion is attributed to a combination of factors, namely calcification, fibrosis, and thrombus formation [1]. Calciphylaxis is a devastating disease known for its high morbidity and mortality rates, where individuals face a challenging six-month survival rate of approximately 50% [1]. Clinically, calciphylaxis manifests as a painful skin condition with varying degrees of intensity, often accompanied by tactile hypersensitivity, skin hardening (induration), the formation of plaques, livedo (mottled, discolored skin), or purpura [2]. In its advanced stages, these initial skin lesions may deteriorate into necrotic eschars, which pose a considerable risk of infection, contributing to an annual mortality rate that ranges from 40% to 80% [3]. The root cause of calciphylaxis lies in calcification, which can result from various sources such as hyperphosphatemia, hypercalcemia, and hyperglycemia. These factors lead to the production and deposition of hydroxyapatite crystals, initiating the pathogenic cascade [3]. The specific underlying cause of calciphylaxis has yet to be definitively established, adding complexity to our understanding of this condition. 

Uremic calciphylaxis has been primarily associated with end-stage renal disease (ESRD). However, emerging evidence from recent case reports suggests that this rare vasculopathy can manifest in the context of early kidney injury, including acute kidney injury (AKI), broadening the scope of its clinical presentation. The diagnostic process for calciphylaxis presents substantial clinical challenges. In patients with established kidney disease, a high degree of clinical suspicion is imperative to identify this disease process. Conversely, in patients without ESRD or those with early kidney injury, calciphylaxis often eludes initial diagnosis. 

Ultimately, the definitive confirmation typically necessitates a skin punch biopsy to access deeper tissue layers [4]. Histopathological examination often reveals distinctive features, such as calcification within the vessels of the dermis and subcutaneous tissue, coupled with evidence of ischemic tissue necrosis. These histological findings differentiate calciphylaxis from other conditions characterized by vascular calcification.

Although there are associated high morbidity and mortality rates, a multifaceted and layered approach to therapy can slow down the progression of the disease.

We seek to portray a unique presentation of calciphylaxis in the setting of AKI, describe clinical findings, and discuss mainstay therapies.

## Case presentation

**Figure 1:**
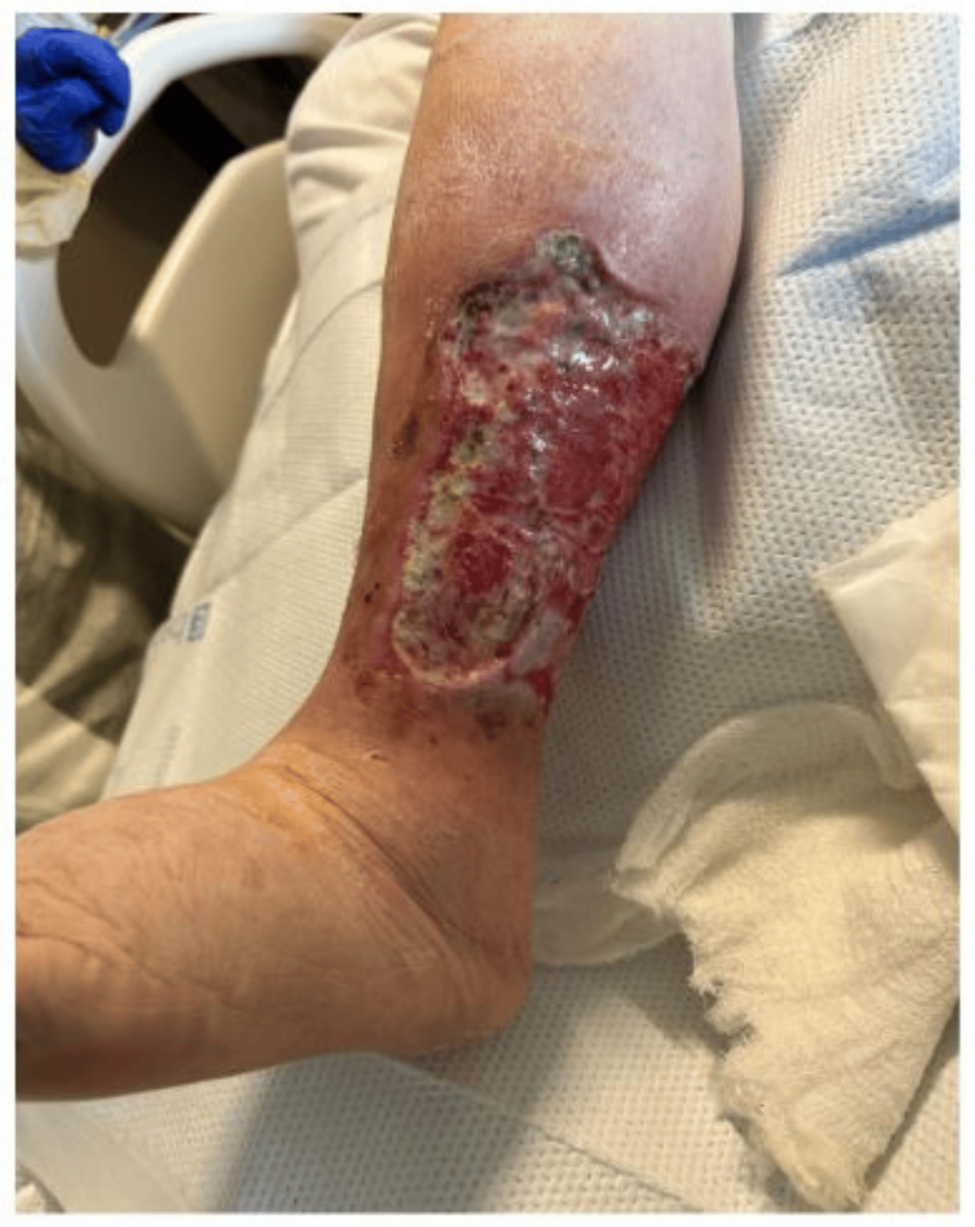
Left lower extremity

**Figure 2:**
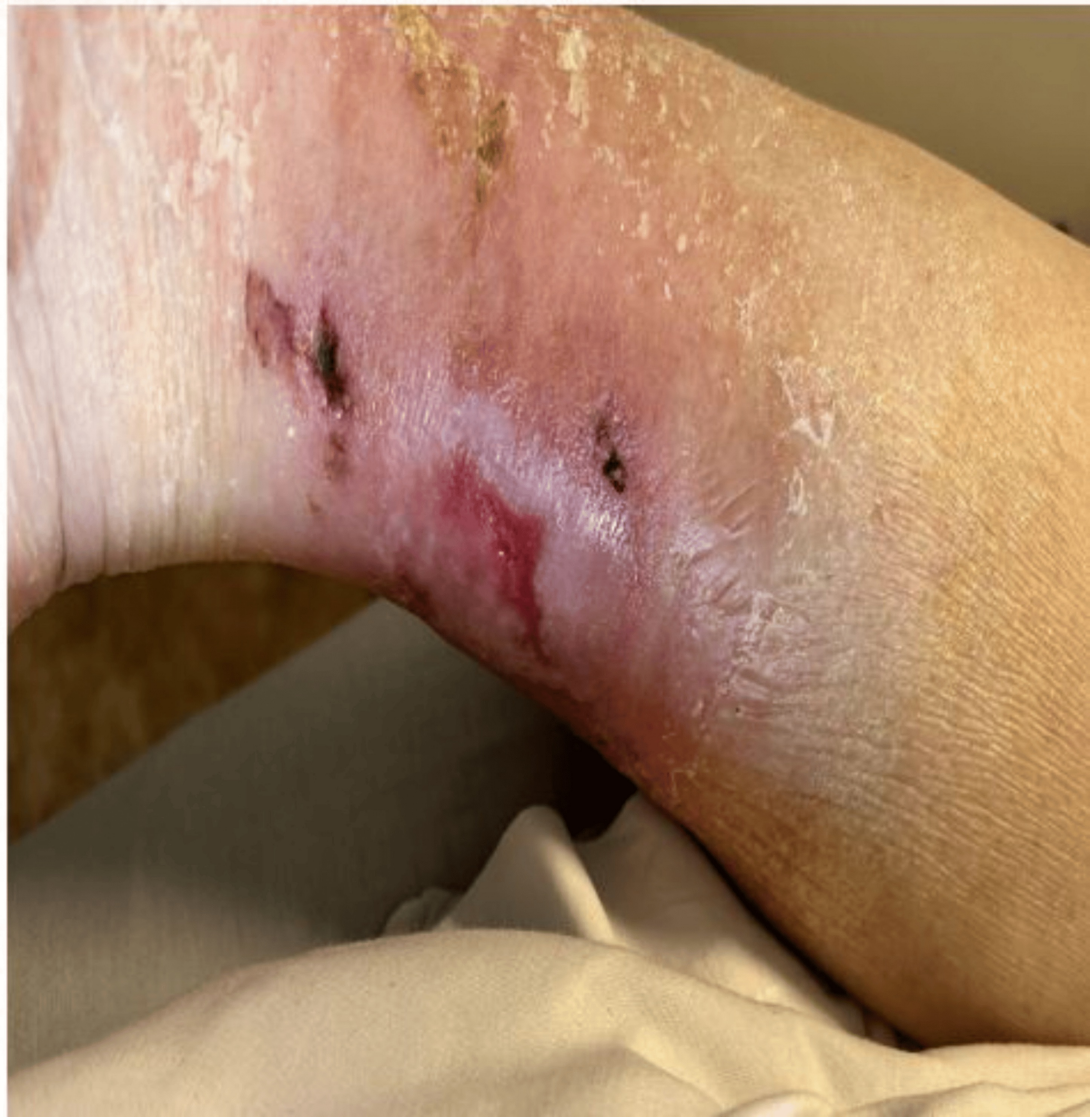
Right lower extremity

A 75-year-old male with a past medical history of hypertension, type 2 diabetes, and recurrent lower extremity wounds presented to the emergency department due to complaints of generalized weakness and worsening bilateral lower extremity wounds for over a month. On a physical exam, the patient was afebrile, with a blood pressure of 109/52, a heart rate of 112 bpm, and oxygen saturation of 98%. He was an age-appropriate male. The patient had an irregular rhythm with a murmur. Lung and abdominal exams were unremarkable. His lower extremities were remarkable for bilateral wounds (14 cm × 14 cm left lower extremity and 3 cm × 3 cm right lower extremity; Figures 1, 2).

**Figure 3:**
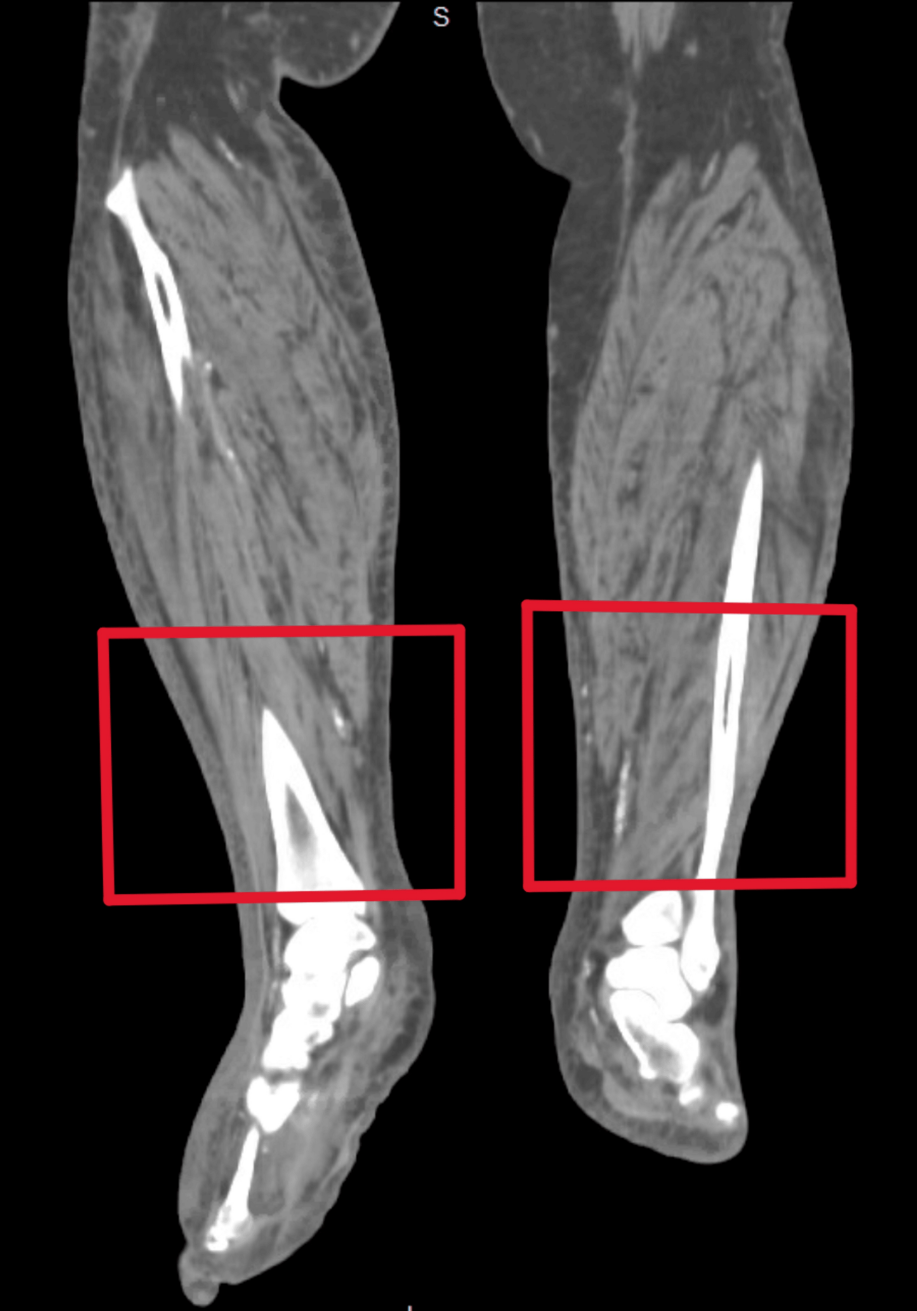
No abscesses (red boxes) notable in the anterior coronal view of bilateral legs

**Figure 4:**
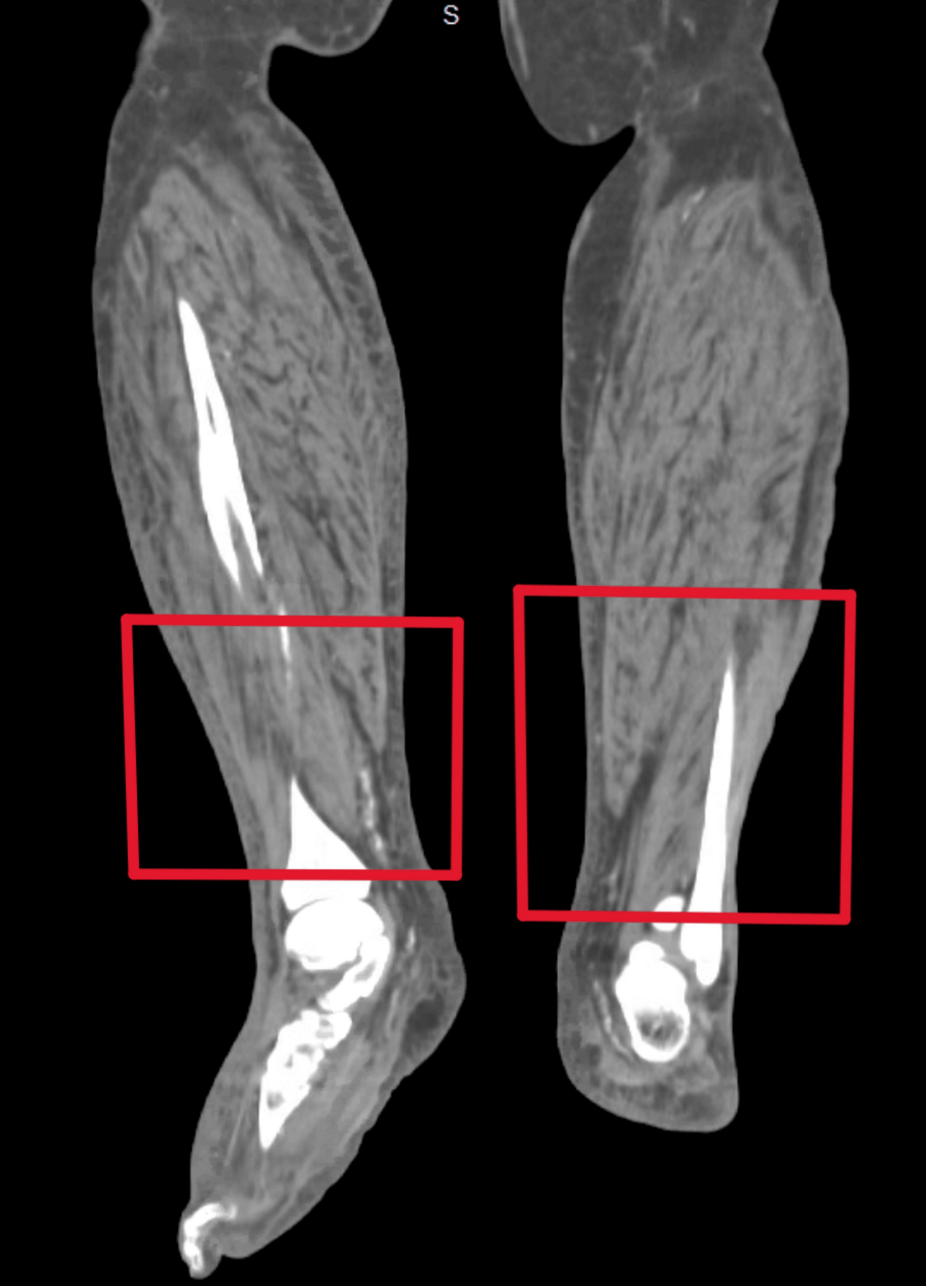
No abscesses (red boxes) notable in the posterior coronal view of bilateral legs

Laboratory testing demonstrated leukocytosis at 15.3 K/mm³, c-reactive protein (CRP) of 23.7 mg/dL, potassium at 7 mmol/L, BUN of 108.9 mg/dL, creatinine of 10.0 mg/dL (baseline creatinine, 1.1 mg/dL), corrected calcium of 10.5 mg/dL, phosphorous level of 7.0 mg/dL, and parathyroid hormone level of 41 pg/mL. CT imaging of bilateral lower extremities showed no abscess formation (Figures 3, 4).

**Figure 5:**
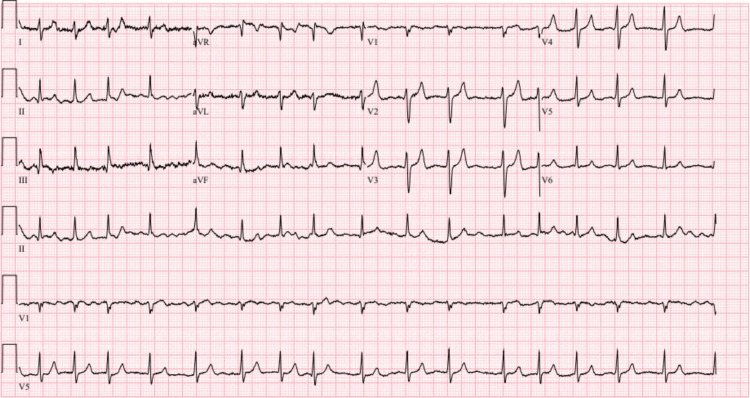
Atrial fibrillation with a heart rate of 100 bpm seen on electrocardiogram

**Figure 6:**
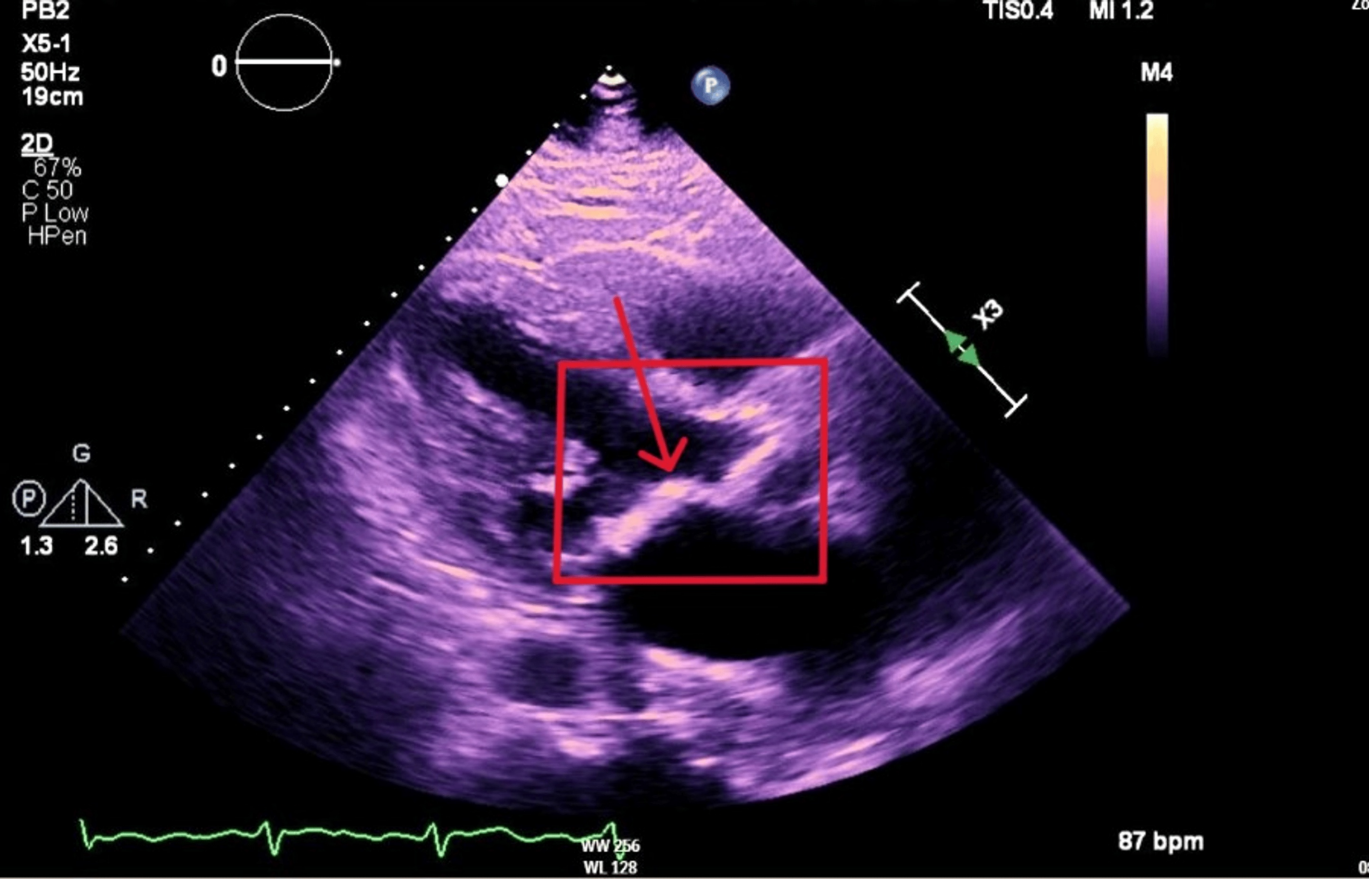
Parasternal view of mitral valve stenosis on echocardiogram (red arrow in red box)

**Figure 7:**
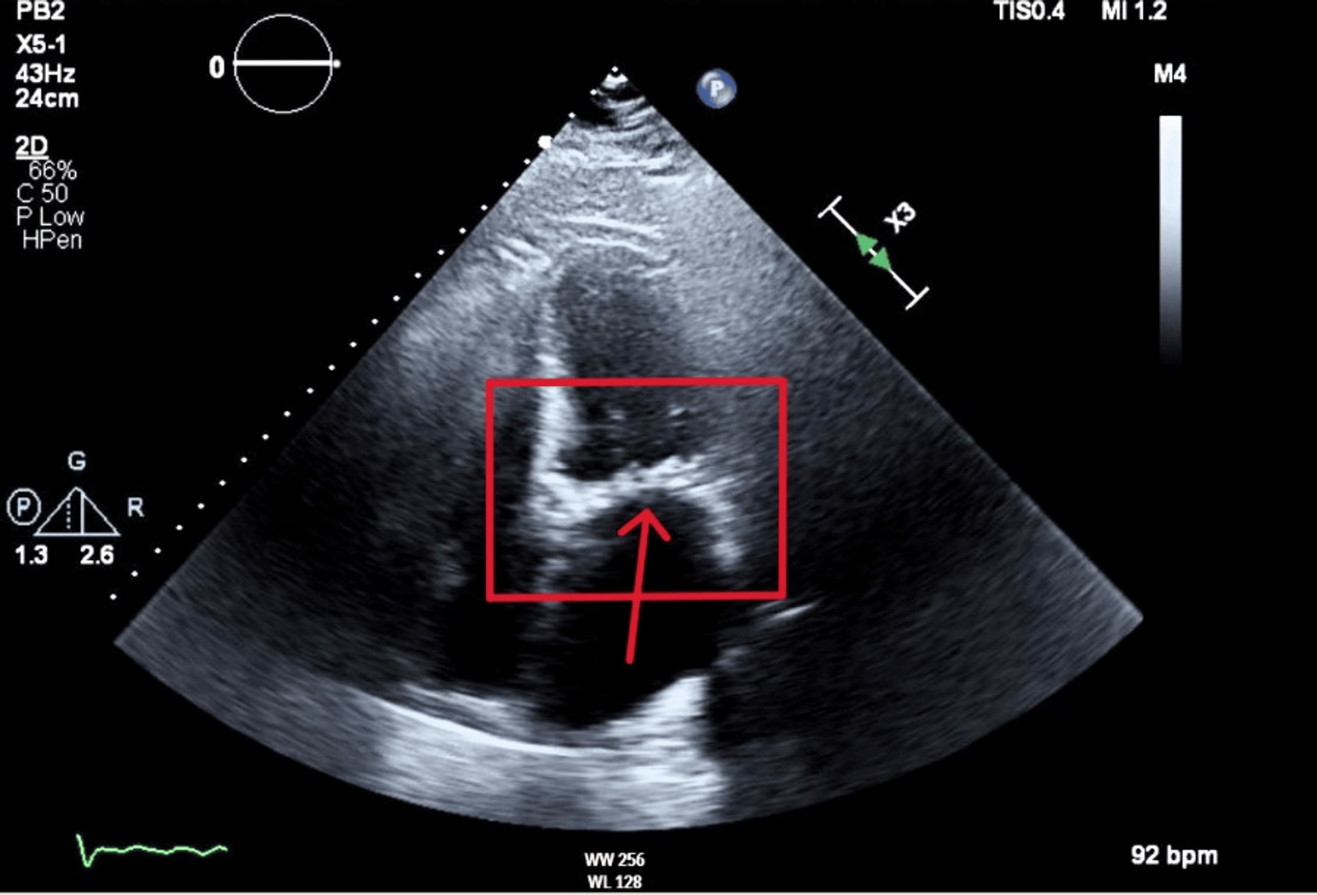
Apical four chamber view of mitral valve stenosis on echocardiogram (red arrow in red box)

Microbiology wound cultures from the leg wounds grew *Acinetobacter lwoffii* and alpha-hemolytic *Streptococcus*. Blood cultures were negative. The patient was admitted to the intensive care unit due to septic shock and was treated with vasopressors and antibiotics. He also underwent one session of hemodialysis. Additionally, the patient was found to have developed new onset atrial fibrillation (Figure 5) and was subsequently started on coumadin due to severe mitral stenosis noted on echocardiogram (Figures 6, 7).

**Figure 8:**
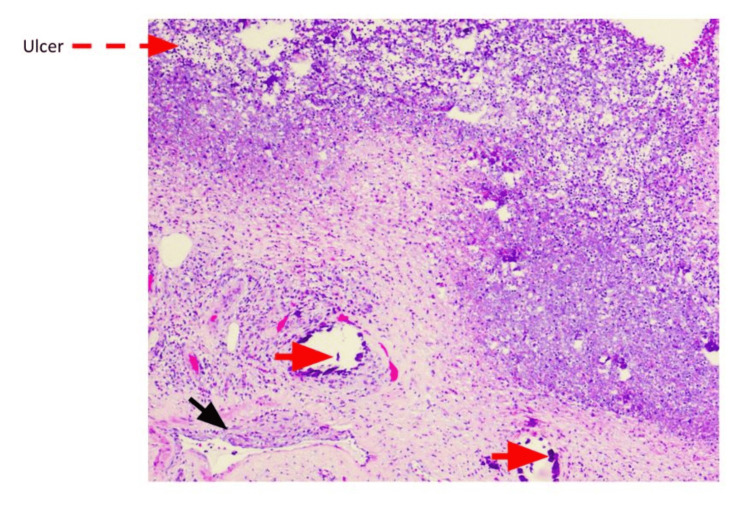
Calciphylaxis: ulcerated and necrotic skin surface (red arrow), dermal small vessel calcifications (red arrowhead), and intimal fibroblastic proliferation with luminal narrowing of vessels (black arrowhead), H&E 4×

**Figure 9:**
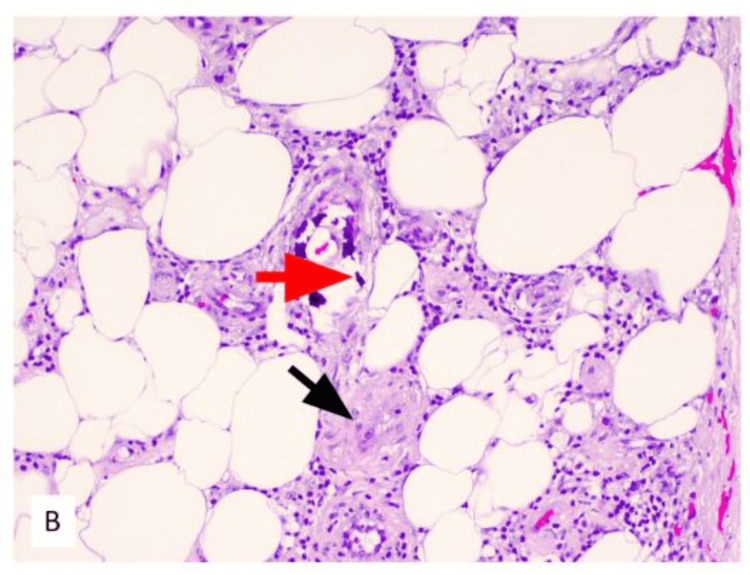
Small vessel calcification (red arrowhead) and intimal fibroblastic proliferation with luminal narrowing within subcutaneous tissue (black arrowhead), H&E 10×

**Figure 10:**
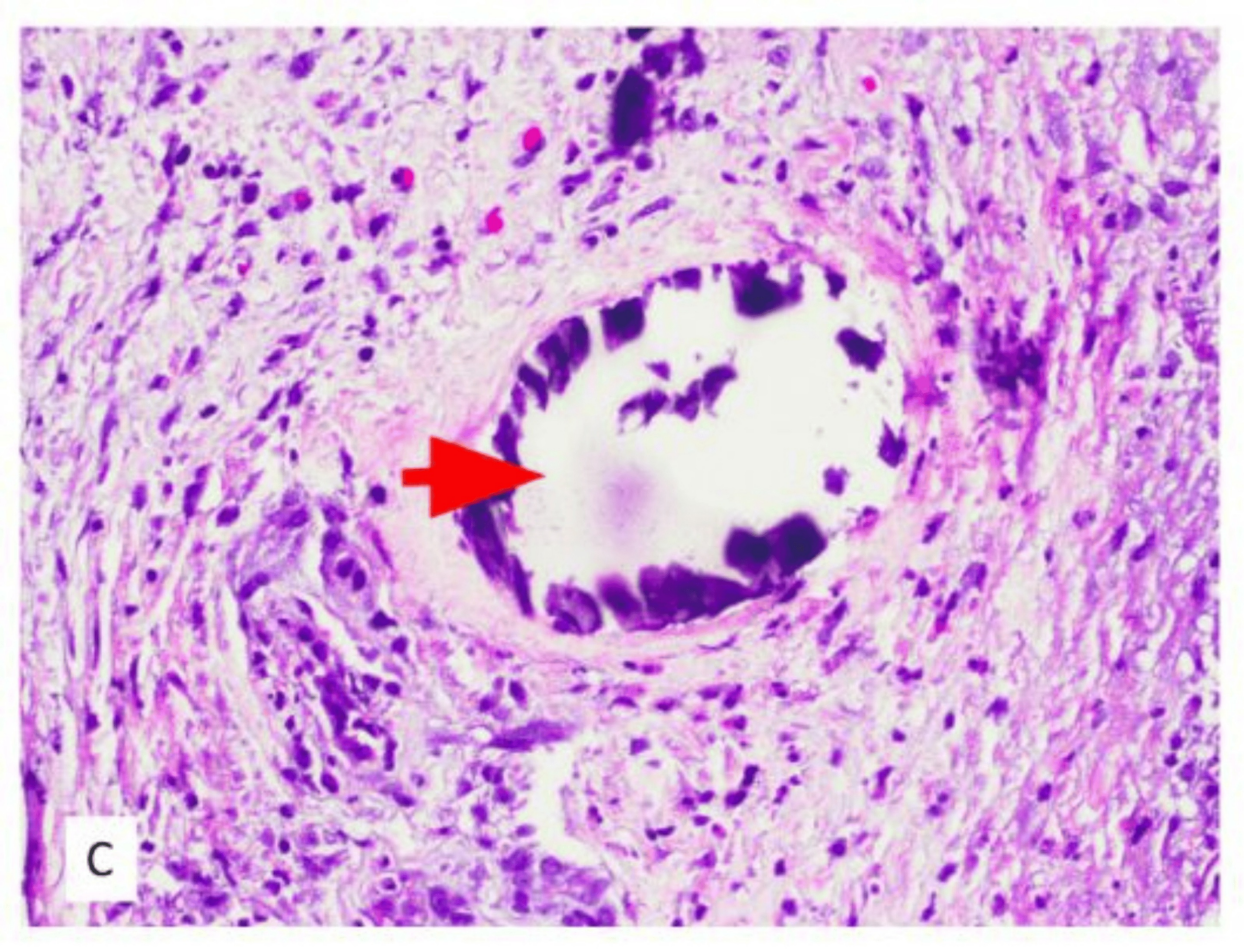
Calcifications within small vessels (red arrowhead), H&E 20×

**Figure 11:**
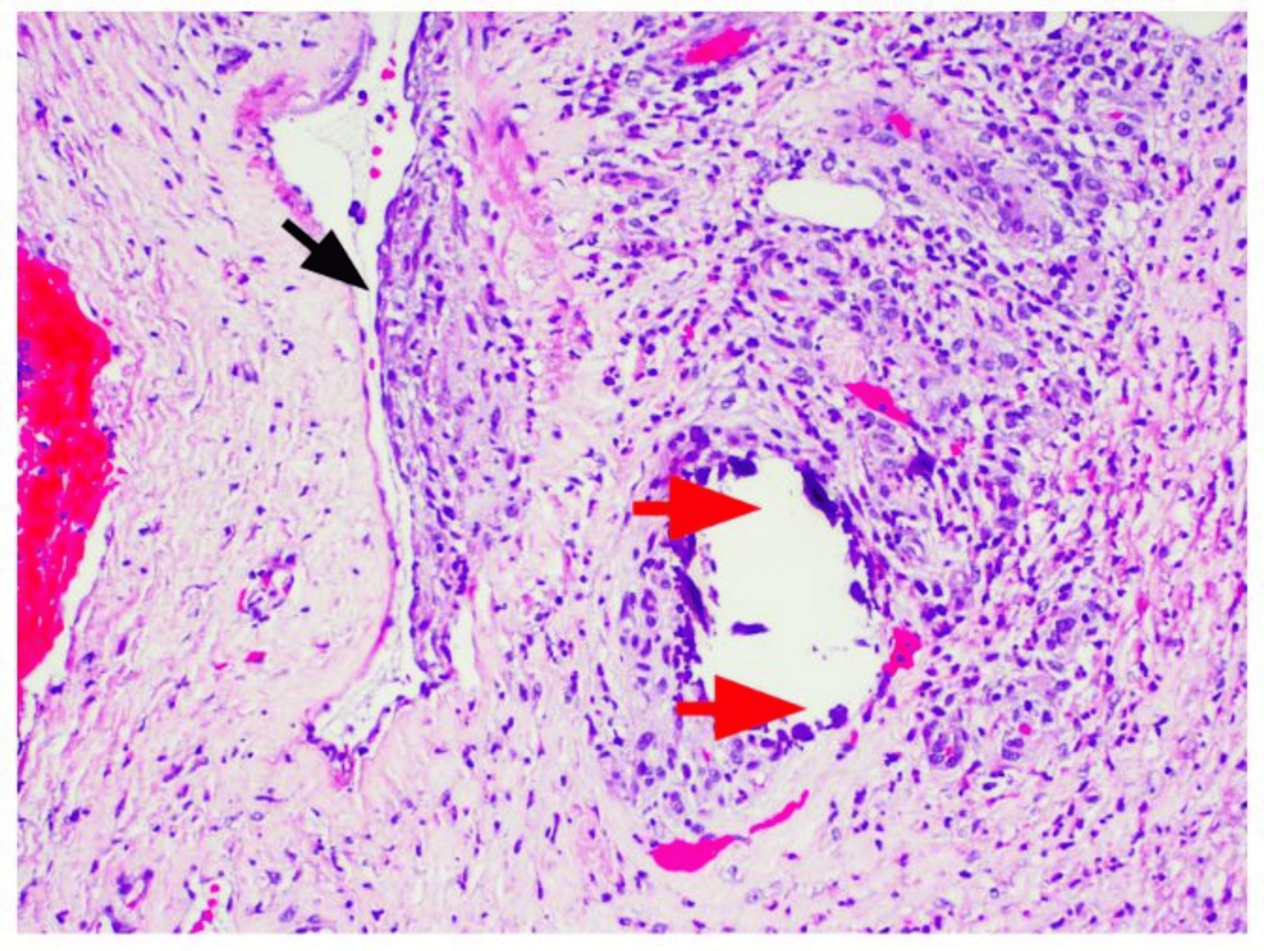
Dermal calcifications (red arrowhead) and intimal fibroblastic proliferation with luminal narrowing of small vessels (black arrowhead), H&E 10×

The patient underwent surgical debridement of the wounds, and the pathology report showed left lower extremity skin and underlying subcutaneous tissue with marked acute inflammation, necrosis, ulceration, and calcification of small- to medium-sized vessels, consistent with calciphylaxis (Figures 8-11). The patient was started on sodium thiosulfate three times per week and was discharged home with close follow-up for his wounds.

## Discussion

Calciphylaxis is not only an extremely debilitating condition that presents with excruciating pain but also found to be life-threatening as the disease worsens. Characterized by calcifications that deposit in microvasculature found in subcutaneous adipose tissue and dermis, these depositions lead to ischemic infarcts that manifest as cutaneous plaques.

Ulceration is the hallmark of calciphylaxis, and the degree of ulceration correlates with the prognosis of the condition [5]. Initially, patients tend to present with skin mottling and induration, similar to that of livedo reticularis, typically localized in the lower extremities, abdomen, and buttocks [6]. Because the blood flow to these regions is compromised, these lesions tend to worsen, developing into plaques, which then progress to ulceration. With only plaques at six months of clinical presentation, there is a 33% mortality rate. However, with the development of ulcers in a six-month clinical presentation, the mortality rate nearly doubles to 67% and can reach as high as 80% after a six-month presentation [5]. Ultimately, the leading cause of death in calciphylaxis is from sepsis, as this population is at risk of infection of these ulcerations [4].

Associated risk factors are thought to be primarily chronic renal failure but also an imbalance in calcium and phosphate homeostasis, vitamin K deficiency and warfarin use, and comorbidities that include obesity, diabetes mellitus, and rapid weight loss [3]. Although the mechanism behind calciphylaxis remains poorly understood, it is believed that the absence of vascular calcification inhibitors is the root cause. As mentioned before, these include fetuin-A, osteoprotegerin, and matrix G1a protein. Fetuin-A binds to calcium and phosphate; therefore, a disturbance in calcium and phosphate homeostasis downregulates fetuin-A. A similar mechanism is seen in patients with ESRD due to vitamin D deficiency. Moreover, vitamin K plays an instrumental role in activating matrix G1a protein, another potent inhibitor of calcification. This protein relies on vitamin K carboxylation for its activation. Thus, an inherent vitamin K deficiency or warfarin use decreased matrix G1a protein activation, increasing the risk of calciphylaxis [7].

Although calciphylaxis has proven to have a negative prognosis, intravenous sodium thiosulfate has proven to be a mainstay in treatment. Sodium thiosulfate acts by chelating calcium salts and forming a more soluble byproduct called calcium thiosulfate. This leads to reducing the amount of calcifications found in adipose and blood vessels [4]. A study conducted at Fresenius Medical Care North America had 172 patients undergoing maintenance hemodialysis receiving a median of 38 doses with a median dose of 25 g. They had notably found calciphylaxis to have resolved in 26.4% of patients, with 18.9% of patients showing marked improvement [8].

Alongside intravenous sodium thiosulfate being a hallmark for treatment, other treatment modalities have assisted in improvement. Firstly, early attention to wound care with appropriate dressings and moisture control assumes paramount importance, as it serves to contain the progression of necrotic tissue and mitigate the risk of infection or subsequent sepsis [3]. Notably, research has shown that early surgical debridement significantly enhances the one-year survival rate, with rates of 61.6% in patients who undergo surgical debridement compared to 27.4% in those who do not [4]. In addition, antibiotics are not recommended but are beneficial if infection is suspected [7]. Lastly, in order to promote further healing, another treatment modality to consider is hyperbaric oxygen therapy as it targets the ischemia present in these wounds.

Until recently, pain control, wound management, and a poor prognosis were all that were offered to these patients. With further understanding of the disease mechanism, using conjunctive sodium thiosulfate has proven to prevent the progression of such a highly devastating disease. 

Our patient’s outpatient records indicated a normal creatinine level. Additionally, given his kidney function improvement to a stable and normal value, it was deduced that he developed calciphylaxis uncharacteristically in the setting of acute renal failure. Moreover, this patient’s course was further complicated by him developing atrial fibrillation in the setting of severe mitral stenosis. In this setting, guidelines recommend warfarin therapy over direct oral anticoagulants as a form of anticoagulation. As discussed earlier, warfarin use becomes a risk factor for developing calciphylaxis as well through its mechanism of impeding vitamin K function. In fact, a study conducted in 2017 notes a case study of warfarin-induced calciphylaxis without the presence of renal injury, concluding that warfarin use in their patient caused calcification of arteries and paradoxical thrombosis through its actions on local vascular endothelium seen similarly in calciphylaxis [9]. However, our patient had been diagnosed with calciphylaxis before introducing warfarin. The clinical decision in this case was difficult due to the presence of severe valvular abnormality. Generally, and in appropriate cases, the use of warfarin should be avoided.

## Conclusions

Calciphylaxis is notable for calcifications that accumulate in microvasculature as well as subcutaneous fat and dermis, leading to vascular as well as dermatologic infarcts. It is well documented that this condition has a clear association with patients in ESRD. However, this particular case highlights a unique presentation of calciphylaxis in a patient with AKI. With new advances in understanding the disease mechanism and new therapeutic agents, identifying disease pathophysiology becomes all that much crucial given the high degree of infection rates and high mortality rates. Therefore, it is important for physicians to keep in mind that in the setting of AKI and the presence of body ulcerations, calciphylaxis should be considered a potential diagnosis.
